# Correction: Zhang et al. Parkin, as a Regulator, Participates in Arsenic Trioxide-Triggered Mitophagy in HeLa Cells. *Curr. Issues Mol. Biol.* 2022, *44*, 2759–2771

**DOI:** 10.3390/cimb45100495

**Published:** 2023-09-26

**Authors:** Zhewen Zhang, Juan Yi, Bei Xie, Jing Chen, Xueyan Zhang, Li Wang, Jingyu Wang, Jinxia Hou, Hulai Wei

**Affiliations:** School of Basic Medical Sciences, Lanzhou University, Lanzhou 730000, China; zhangzhw@lzu.edu.cn (Z.Z.); yij@lzu.edu.cn (J.Y.); xieb@lzu.edu.cn (B.X.); zhangxy@lzu.edu.cn (X.Z.); wangli15@lzu.edu.cn (L.W.); houjx17@lzu.edu.cn (J.H.)

## Correct Email Address

In the published publication [1], there were some errors regarding the email addresses for Wang Jingyu and Hou Jinxia. The correct email addresses should be wangjingyu@lzu.edu.cn and houjx17@lzu.edu.cn.

## Figure Correction

In the original publication [1], there was a mistake in Figure 5A in the published paper. YFP parkin cells are HeLa cells with YFP Parkin gene transferred. There is no difference in morphology between YFP parkin cells and parent HeLa cells. As a result, the photos were confused during the screenshot operation on the original image, resulting in the misplacement of the photos. The corrected Figure 5A appears below. The authors state that the scientific conclusions are unaffected. This correction was approved by the Academic Editor. The original publication has also been updated.

## Figures and Tables

**Figure 5:**
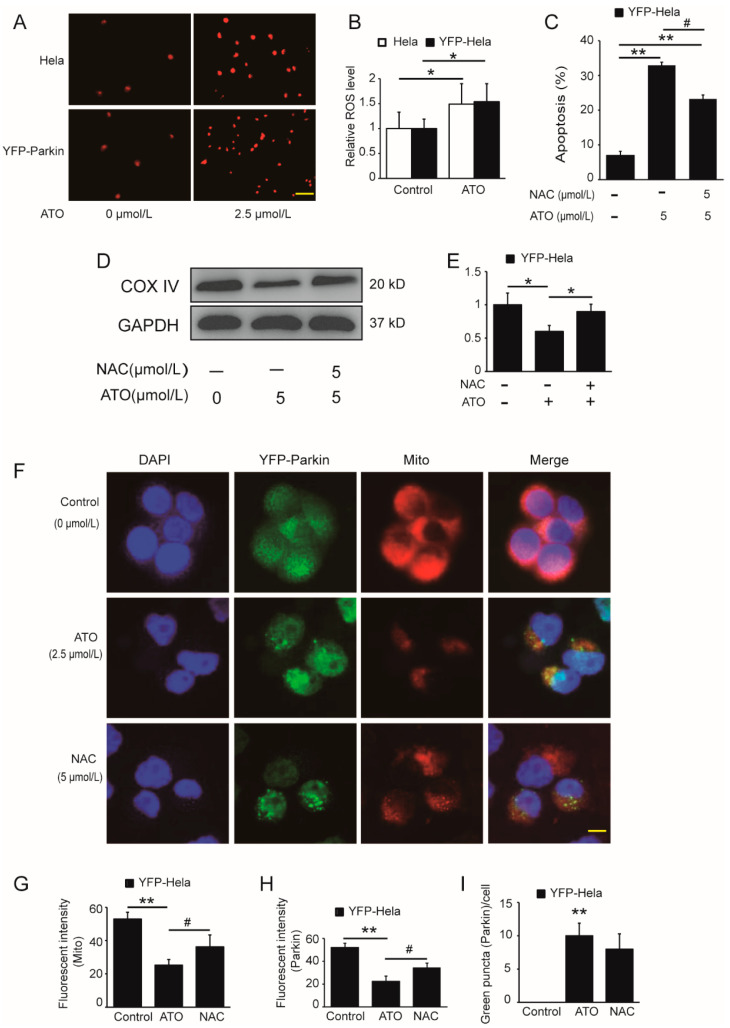
Potentiating effect of ATO on ROS production. (**A**) Red fluorescence indicates the production of ROS after cell treatment for 6 h, and the images were analyzed using a fluorescence microscopy. Scale bar, 10 μm. (**B**) shows the quantification of the red fluorescence, which was performed using Image J software. (**C**) YFP-Parkin HeLa cells were treated with the indicated concentrations of ATO for 24 h before detection of cell apoptosis by flow cytometry. (**D**) Western blot analysis of COX IV expression rescued after ATO treatment for 24 h in YFP-Parkin HeLa cells. (**E**) Quantification of D. (**F**) YFP-Parkin HeLa cells were fixed and immunostained for nuclei (blue) and mitochondria (red) after ATO treatment for 24 h. The red fluorescence indicated the normal mitochondrial membrane potential. The green fluorescence was the marker of YFP-Parkin proteins. The level of mitophagy was evaluated by the number of green puncta. The samples were analyzed using a fluorescence microscopy. Scale bar, 10 μm. (**G**) Quantification of the red fluorescence, which was performed using Image J software. (**H**) Quantification of the green fluorescence, which was performed using Image J software. (**I**) Quantification of green puncta, which was performed using Image J software. The histograms represent the mean ± S.D. of triplicate experiments. * *p* < 0.05 vs. 0 μmol/L group, ** *p* < 0.01 vs. 0 μmol/L group, # *p* < 0.05 vs. without Parkin group. Without NAC group; GAPDH served as an internal control.
